# A common missense variant of *LILRB5* is associated with statin intolerance and myalgia

**DOI:** 10.1093/eurheartj/ehx467

**Published:** 2017-08-29

**Authors:** Moneeza K Siddiqui, Cyrielle Maroteau, Abirami Veluchamy, Aleksi Tornio, Roger Tavendale, Fiona Carr, Ngu-Uma Abelega, Dan Carr, Katyrzyna Bloch, Par Hallberg, Qun-Ying Yue, Ewan R Pearson, Helen M Colhoun, Andrew D Morris, Eleanor Dow, Jacob George, Munir Pirmohamed, Paul M Ridker, Alex S F Doney, Ana Alfirevic, Mia Wadelius, Anke-Hilse Maitland-van der Zee, Daniel I Chasman, Colin N A Palmer, PREDICTION-ADR Consortium

**Affiliations:** 1 Pat McPherson Centre for Pharmacogenetics & Pharmacogenomics, Division of Molecular & Clinical Medicine, University of Dundee, Ninewells Hospital and Medical School, Dundee DD19SY, UK; 2 Institute of Translation Medicine, University of Liverpool, Liverpool L69 3BX, UK; 3 Department of Medical Sciences, Clinical Pharmacology and Science of Life Laboratory, Uppsala University, 751 85 Uppsala, Sweden; 4 Medical Products Agency, Dag Hammarskjölds väg 42, 75237 Uppsala, Sweden; 5 Institute of Genetics & Molecular Medicine, University of Edinburgh, Edinburgh EH4 2XU, UK; 6 Usher Institute of Population Health Sciences and Informatics, University of Edinburgh, Edinburgh EH4 2XU, UK; 7 Ninewells Hospital and Medical School, Dundee DD19SY, UK; 8 Brigham and Women's Hospital, Department of Medicine, Preventive Medicine, Harvard Medical School, 75 Francis Street, Boston, MA 02115, USA; 9 Division of Pharmacoepidemiology and Clinical Pharmacology, Utrecht Institute of Pharmaceutical Sciences, Utrecht University, 3508 TB Utrecht, The Netherlands; 10 Department of Respiratory Medicine, Academic Medical Center, University of Amsterdam, Meibergdreef 9, 1105 AZ Amsterdam, The Netherlands

**Keywords:** Statins, Pharmacogenetics, Immunogenetics, Precision medicine, Adverse drug reactions, Myalgia

## Abstract

**Aims:**

A genetic variant in *LILRB5* (leukocyte immunoglobulin-like receptor subfamily-B) (rs12975366: T > C: Asp247Gly) has been reported to be associated with lower creatine phosphokinase (CK) and lactate dehydrogenase (LDH) levels. Both biomarkers are released from injured muscle tissue, making this variant a potential candidate for susceptibility to muscle-related symptoms. We examined the association of this variant with statin intolerance ascertained from electronic medical records in the GoDARTS study.

**Methods and results:**

In the GoDARTS cohort, the *LILRB5* Asp247 variant was associated with statin intolerance (SI) phenotypes; one defined as having raised CK and being non-adherent to therapy [odds ratio (OR) 1.81; 95% confidence interval (CI): 1.34–2.45] and the other as being intolerant to the lowest approved dose of a statin before being switched to two or more other statins (OR 1.36; 95% CI: 1.07–1.73). Those homozygous for Asp247 had increased odds of developing both definitions of intolerance. Importantly the second definition did not rely on CK elevations. These results were replicated in adjudicated cases of statin-induced myopathy in the PREDICTION-ADR consortium (OR1.48; 95% CI: 1.05–2.10) and for the development of myalgia in the JUPITER randomized clinical trial of rosuvastatin (OR1.35, 95% CI: 1.10–1.68). A meta-analysis across the studies showed a consistent association between Asp247Gly and outcomes associated with SI (OR1.34; 95% CI: 1.16–1.54).

**Conclusion:**

This study presents a novel immunogenetic factor associated with statin intolerance, an important risk factor for cardiovascular outcomes. The results suggest that true statin-induced myalgia and non-specific myalgia are distinct, with a potential role for the immune system in their development. We identify a genetic group that is more likely to be intolerant to their statins.

## Introduction

Statins are first choice lipid-modifying medications for prevention and management of cardiovascular diseases (CVD).^1^^,^^2^ The UK is one of the largest users of statins worldwide,^3^ and with revised NICE guidelines approximately 12 million UK individuals will be prescribed statins.^4^^,^^5^ While statins are generally well tolerated, neurological,^6^ gastro-intestinal, or muscle-based^7^^,^^8^ adverse drug reactions are reported. Adverse reactions to statins are likely to manifest as muscle aches (myalgia) along with elevated creatine phosphokinase (CK). Adherence to statin treatment is often negatively impacted in response to adverse reactions.^9^^,^^10^ The inability to adhere to statin treatment, whether due to statin-induced myalgia or more general forms of statin intolerance result in poor on-statin outcomes.^11^ Therefore, examining risk factors predisposing to statin intolerance is crucial from a public health perspective.

A genome-wide association study (GWAS) by Dubé *et al.*,^12^ reported a missense variant Asp247Gly in the leukocyte immunoglobulin-like receptor subfamily B member 5 gene, *LILRB5* on chromosome 19, base position 54759361 (Human Genome Build GRCh37), was associated with circulating serum CK levels. The mean CK levels of Asp247 homozygotes (T/T) were significantly higher. This association was found to be independent of statin use, however there is no known biological mechanism for the variant in determining CK levels. A GWAS by Kristjansson *et al.*^13^ of over 60 000 Icelanders replicated the association of the variant and CK levels. The same study also reported the association of the variant with serum lactate dehydrogenase (LDH) levels in a population of over 90 000 Icelanders.^13^ The *LILRB5* variant showed the same direction of effect, i.e. Asp247 homozygotes had higher LDH and CK levels. LDH is often used in conjunction with CK as a marker of tissue damage. The findings suggest the variant might impart a statin independent susceptibility to muscle-based events. This makes *LILRB5* a potential marker for susceptibility to the commonly noted muscle-based symptoms attributed to statin intolerance.

These discoveries warrant an investigation into the role of the *LILRB5* variant in statin intolerance. Population-based studies use surrogate markers of intolerance, such as elevations in CK, trends in statin treatment, dose reductions, switching or the discontinuation of therapy. Therefore, *a priori*, we considered two definitions of statin intolerance, one dependent on and one independent of elevated CK levels. We hypothesize that carriers of variant associated with higher muscle enzyme levels (CK and LDH) will also be predisposed to forms of statin intolerance independent of CK levels.

The principal cohort used was the Genetics of Diabetes Audit and Research, Tayside Scotland (GoDARTS). GoDARTS has been previously used to establish pharmacogenetic associations of genes such as the hepatic influx transporter *SLCO1B1* and statin intolerance.^14^ At present, GoDARTS contains 11 912 statin users and provides approximately 98 000 person-years of statin exposure, providing an ideal cohort to examine the association of this genetic variant with statin intolerance. Replication was examined in the Clinical Practice Research Datalink (CPRD) STAGE study^15^ and clinically adjudicated cases of statin-induced myopathy (SIM) in the European PREDICTION-ADR consortium study.^9^ The interaction of this effect with statin use was then studied among participants who developed myalgia in the JUPITER (Justification for the Use of Statins in Prevention: an Intervention Trial Evaluating Rosuvastatin) randomized clinical trial (RCT) where individuals were allocated rosuvastatin or placebo to assess the relative reduction in vascular events.^16^^,^^17^

## Methods

### GoDARTS cohort

#### Study population

Tayside Medical Ethics Committee approved the GoDARTS study and informed consent was obtained for all participants. The dataset contains complete electronic medical records (EMR), prescription information and laboratory results from 18 306 Scottish Caucasian individuals. Prescribing data were available from 1 January 1990 to 31 July 2013. In all, 10 149 study participants had type 2 diabetes (T2D) at recruitment and the remainder (8157) were recruited as non-diabetic controls. We performed a case-control study for statin intolerance in this population. We found 11 947 statin users from GoDARTS who had at least two prescriptions of statins. The prescription patterns indicating intolerance used in this study are similar to those used by Donnelly *et al.*^14^ to establish the association between statin intolerance and *SLCO1B1* genotypes in the GoDARTS study. For features used to define statin intolerance and tolerance, see Supplementary Material online, *Methods S1*.

### Statin intolerance in GoDARTS

#### General statin intolerance

Cases of general statin intolerance (GSI) were defined as users with CK raised above the ULN (Upper Limit of Normal), after start of statin therapy, who either switched statin therapies two or more times (not including systemic shifts to atorvastatin after patent expiration in the UK) or discontinued therapy (*n* = 588). Controls (statin tolerant individuals—ST1) had over 90% coverage with statin prescriptions, for a minimum of 5 years, at a minimum average daily dose of 40 mg of simvastatin (or equivalent dose of another statin), had consistently normal CKs while on statins, had never switched their statin therapy (except the systematic switch to atorvastatin) and had not discontinued therapy (*n* = 356).

#### Lowest approved daily starting dose statin intolerance

Since the *LILRB5* Asp247Gly variant was known to be associated with CK levels, a phenotype independent of CK elevations was created in order to determine if the association of the variant with statin intolerance was confounded by the variant‘s association with CK levels. The European Society of Cardiology and European Atherosclerosis Society joint recommendations on the management of dyslipidaemias suggest cessation of statin treatment if the user presents with normal CK, but persistent symptoms of intolerance.^18^ This intolerance definition was derived from the GAUSS-2 trial, the consensus definition based on recommendations by Banach *et al.* and recommendations of the National Lipid Association (NLA) in 2014.^19–22^

Cases of low dose intolerance (LDI) had used two or more different statins, and at least one statin that was discontinued would have to be at the lowest approved daily starting dose (NLA, 2014) before discontinuation, irrespective of their CK levels (*n* = 591). Controls (statin tolerant individuals—ST2) met all the criteria of the previous statin tolerant (ST1) group, except the definition was independent of the CK elevation criteria (*n* = 443). A higher proportion of controls had CK levels in the normal range, specifically, 354 of the 443 (80%) controls compared to 335 of 591 (57%) cases. Therefore, association tests for this phenotype were adjusted for (log-transformed) CK levels, in addition to other covariates.

For genotyping methods, see Supplementary material online, *Methods S1*.

#### Validation of statin intolerance phenotypes in GoDARTS

These phenotypic definitions of statin intolerance were validated against known *SLCO1B1* genotype risk score^14^ and the outcome of major adverse cardiovascular event (see Supplementary material online, *Methods S2*). They were significantly associated with both.

### Replication studies

#### CPRD-STAGE study: statin-induced myopathy

Replication was sought from the CPRD-STAGE study.^15^ Data were available for 129 cases of SIM and 2501 population controls from the Wellcome Trust Case Control Consortium (WTCCC1).^15^ Cases of statin myopathy were identified using CPRD and tertiary muscle clinics and conformed to SIM classification standards.^9^ Analysis presented is unadjusted for covariates due lack of available data for the WTCCC population controls. For additional cohort information, see Supplementary material online, *Methods S3*.

#### PREDICTION-ADR: statin-induced myopathy

Cases and controls for SIM were contributed by the consortium‘s study centres in Uppsala (Sweden), Dundee and Liverpool (UK). Cases met criteria for classification of SIM.^9^ Identification of SIM from population cohorts: GoDARTS, Genetics of Scottish Health Registry (GoSHARE)^23^ and CPRD using EMRs was based on on-statin CK levels raised ≥4 times ULN. Subsequently, clinical adjudication was undertaken by physicians and specialists. Factors considered were resolution of CK after de-challenge, post-event prescribing changes (e.g. switching or total discontinuation), medical history of kidney disease, trauma, falls, myocardial infarction, thyroid disease and tests for HMGCR (3-Hydroxy-3-Methylglutaryl-CoA Reductase) antibodies, muscle biopsy and physical activity, if available. Cases were also identified from CVD clinics, general practitioners (GP) practices, and muscle disease clinics where adjudication was performed directly by physicians. Swedish cases were selected from Swedegene Biobank, which recruits patients reported to the adverse drug reaction registry at Medical Products Agency, Uppsala. Statin tolerant controls were on therapy for a minimum of 1 year with no recorded adverse events. Analysis was performed on 229 cases of SIM and 432 adjudicated controls of Caucasian ethnicity. Whole-exome sequencing was undertaken in laboratories in Liverpool, Dundee and Uppsala. For details of sequencing methods, see Supplementary material online, *Methods S4*. There were no sample overlaps from GoDARTS in the discovery and replication cohorts.

#### JUPITER trial: myalgia

The replication in JUPITER focused on 8749 study participants of verified European ancestry with available genetic data among whom 4381 were randomized to receive statin treatment and 4368 were randomized to placebo. The population demographics of the genotyped sub-population of the JUPITER trial has been previously described.^16^ The median follow-up period in the trial was 1.9 years, during which traits such as CK, therapy compliance, and myalgia were recorded.^17^ Myalgia was ascertained by physicians blinded to trial-allocation arm^24^ and 837 participants in the study sample were recorded as developing myalgia. Due to possible association between CK measures and diagnosis of myalgia, log-transformed final CK levels were included in analyses as a potential confounder. For genotyping methods, see Supplementary material online, *Methods S1*.

### Statistical analyses

**Figure 1:**
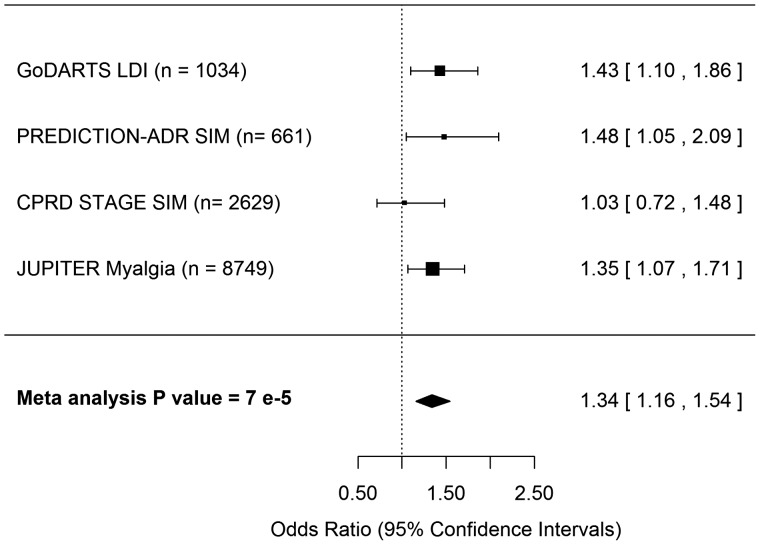
Forest plot representing meta-analysis of the association between *LILRB5* Asp247Gly and outcomes observed across GoDARTS, CPRD-STAGE, PREDICTION-ADR, and JUPITER studies. Study sample size is in parentheses. LDI, low-dose intolerance; SIM, statin-induced myopathy.

All statistical analyses on GoDARTS, CPRD-STAGE, and PREDICTION-ADR data were performed in SAS 9.3 (SAS Institute, Cary, NC, USA). Statistical analyses in JUPITER were performed using R.^25^ Binary logistic regression was used to test the association between the variant and each phenotype of intolerance. Covariates associated with intolerance such as gender, age, co-medication usage, type of statin, dose of statin, and CK levels were added to models where appropriate and available. A backwards step-wise approach was used to eliminate covariates that were not significant predictors in adjusted models. Finally, a fixed-effects meta-analysis on results from the discovery and replication cohorts was performed. Only one phenotype from GoDARTS could be selected since the two groups contained overlapping individuals. LDI phenotype was selected as the phenotype definition did not include the CK levels and the model was adjusted for CK measures. The analysis was performed using the metafor package in R^26^ and results are presented in a Forest plot (*Figure 1*).

The minor allele frequency in GoDARTS, CPRD, PREDICTION-ADR, and JUPITER study populations were 0.37, 0.39, 0.37, and 0.40, respectively. The effect of the *LILRB5* variant was considered dominant based on large-scale analyses with serum CK in GoDARTS (see Supplementary material online, *Results S1*). Therefore all association tests compared those homozygous for Asp247 (T/T) with carriers of 247GlyX (T/C or C/C).

## Results

### Baseline characteristics of general statin intolerance and low dose intolerance

Covariates associated with statin use or with the development of adverse drug reactions (ADR) were tested. Specifically, mean age at start and end of statin therapy, sex, diagnosis of T2D, first and last statin used, starting and ending doses, use of interacting co-medications, statin use for the secondary prevention of CVD, CK levels, and LDL levels prior to statin use. The Comparison of GSI with statin tolerance (ST1) and of LDI with statin tolerance (ST2) is presented (*Table 1*).

**Table 1:** Contrasting general statin intolerance with raised CK and statin tolerance (ST1) and low dose intolerance with statin tolerance (ST2)

Variables	GSI		*P-*value	LDI		*P-*value
	Cases (*n* = 588)	Controls (*n* = 356)		Cases (*n* = 591)	Controls (*n* = 443)	
Mean age start therapy (SD)	60 (10)	62 (10)	0.005	60 (10)	60 (10)	0.9
Years on statin therapy (SD)	10.4 (5)	9.3 (3)	<0.0001	10 (5)	9.5 (3)	0.007
Sex (% females)	50	43	0.007	48	46	0.16
Type 2 diabetics (%)	78	79	0.68	92	90	0.33
First statin as simvastatin (%)	64	71	<0.0001	59	65	<0.0001
Last statin as simvastatin (%)	31	41	<0.0001	31	36	<0.0001
Starting dose as ‘low’ (<20 mg/day) (%)	85	53	<0.0001	94	37	<0.0001
Ending dose as ‘high’ (≥80 mg/day) (%)	22	36	<0.0001	23	50	<0.0001
Interacting co-medications (yes %)	52	44	0.0025	51%	42%	<0.0001
Statin use for secondary prevention of CVD (%)	27	23	0.18	28%	25%	0.3
CK levels (IU/L)^a^						
Median	200	76	<0.0001	98	85	<0.0001
Mean (minimum, maximum)	306 (120, 12, 700)	81 (17, 179)		170 (13, 12 735)	107 (19, 1369)	
LDL levels (mmol/L)^a^						
Median	3.5	3.2	0.07	3.2	3.1	0.38
Mean (minimum, maximum)	(3.5) (1.1, 5.5)	3.2 (0.4, 8.7)		3.1 (1.1, 6.4)	3.2 (0.5, 8.7)	

a Indicates associations were tested using log 10 transformed values.

SD, standard deviation; GSI, general statin intolerance; LDI, Low dose intolerance.

Covariates associated with GSI included younger age at start and longer duration of therapy, female gender, lower incidence of simvastatin use at the start and end of therapy, lower starting and ending dose, the use of interacting co-medications and, by phenotype definition, CK levels at time of diagnosis.

Covariates associated with LDI included longer duration of statin therapy, lower frequency of simvastatin use at the start and end of therapy, lower starting and ending dose, the use of interacting co-medications and CK levels. A concern with a phenotype that is dose dependent is that differences might arise from LDL cholesterol reduction required between tolerant and intolerant individuals, however LDL levels prior to statin therapy were not significantly different across the groups.

### Association between statin intolerance and the *LILRB5* Asp247Gly variant in GODARTS

The variant effect was observed in both unadjusted and adjusted models for both phenotypes (*Table 2*). Individuals homozygous for Asp247 had 1.96 [(95% confidence interval (CI): 1.25–3.07] times the odds of having GSI compared with carriers of the 247Gly variant, in a model adjusted for first statin on therapy, dose, age, sex, and concurrent use of interacting medications (*Table 2*). For LDI, individuals homozygous for *LILRB5* Asp247 genotype had 1.43 (95% CI: 1.10–1.86) times the odds of being intolerant compared to carriers of the 247Gly variant, in a model adjusted for the first and last statin on therapy, CK levels and concurrent use of interacting medications (*Table 2*).

**Table 2:** Association of phenotypes of statin intolerance with *LILRB5* Asp247Gly

Statin intolerance phenotype description	*LILRB5*			
	Asp247Gly (T/C)			
	Unadjusted model		Adjusted model	
	Asp247Asp vs. Gly247X: OR (95% CI)	*P*-value	Asp247Asp vs. Gly247X: OR (95% CI)	*P*-value
GSI	1.62 (1.24, 2.12)	4 × 10^−4^	1.96 (1.25–3.07)	3 × 10^−3^
LDI	1.36 (1.07, 1.73)	0.013	1.43 (1.10–1.86)	7 × 10^−3^

Odds of intolerance for those homozygous for the ancestral allele (Asp247: T/T) are being contrasted to carriers of the minor allele (Gly247X: T/C or C/C) first in main effects or unadjusted models and later in models adjusted for significant covariates. For GSI, the covariates were first statin on therapy, dose of the statin, age, sex and concurrent use of interacting medications. For LDI, the covariates were the first and last statin while on therapy, CK levels and concurrent use of interacting medications.

GSI, general statin intolerance; LDI, Low dose intolerance; CK, creatine phosphokinase; OR, odds ratio; CI, confidence interval.

### Results of replication studies

Replication of the genotypic effect was noted in two out of three studies of statin-related adverse outcomes. In the CPRD-STAGE study, the association was found to be non-significant [odds ratio (OR) 1.03, 95% CI: 0.72–1.49] in a main effects model. However, in the PREDICTION-ADR cohort of SIM, individuals homozygous for *LILRB5* Asp247 had higher odds of developing SIM (OR 1.48, 95% CI: 1.02–2.10) compared to those carrying the 247Gly variant (*Table 3*). Cases and controls were matched for sex, type of statin at time of event during recruitment. The analysis was adjusted for study centre.

**Table 3:** Distribution of *LILRB5* Asp247Gly genotypes by clinically adjudicated SIM status

Group	Asp247Asp (T/T)	Gly247X (T/C or C/C)	Total	Association
Statin-induced myopathy	96	133	229	Odds ratio = 1.48
Statin tolerant controls	161	271	432	95% CI: 1.05–2.10
Total	257	404	661	*P-*value 0.025

Cases and controls were selected from PREDICTION-ADR in Dundee, Liverpool and Uppsala. Model was adjusted for study center.

CI, confidence interval.

In the JUPITER trial, *LILRB5* Asp247Gly had a statistically significant effect on development of myalgia. In a model adjusted for final CK levels and treatment allocation arm, individuals homozygous for Asp247 had 1.35 (95% CI: 1.07–1.71) times the odds of developing myalgia compared to 247Gly variant carriers (*Table 4*). Interestingly, interaction between genotype and statin use was significant (*P-*value 0.04). An analysis of effect stratified by genotype (*Table 5*), showed that a statin-specific myalgia effect is only seen among 247Gly variant carriers. Therefore, while Asp247 homozygotes have an overall higher risk of myalgia, statin-induced myalgia is only observed in 247Gly variant carriers. Similar results are observed in survival analysis (see Supplementary material online, *Results S2*).

**Table 4:** Odds ratios of developing myalgia in the JUPITER trial

Variables	*β*	Odds ratio 95% confidence interval	*P-*value
Main effects model			
Asp247Asp vs. 247GlyX Gly247X	0.16	1.17 (1.02–1.35)	0.03
Adjusted model			
Asp247Asp vs. 247GlyX	0.30	1.35 (1.07–1.71)	0.01
Rosuvastatin vs. placebo	−0.13	0.88 (0.68–1.13)	0.34
Final CK measure (log transformed)	0.19	1.21 (0.87–1.69)	0.26
Asp247Gly*rosuvastatin	−0.34	0.71 (0.51–0.99)	0.04

The adjusted model accounts for treatment allocation arm and final CK measures.

CK, creatine phosphokinase.

**Table 5:** Association of rosuvastatin treatment with myalgia stratified by genotype in the JUPITER trial

Comparison groups	Hazards ratio (95% CI)	*P*-value
Asp247 (T/T)		
Rosuvastatin vs. placebo	0.87 (0.67–1.12)	0.31
247GlyX (C/T or C/C)		
Rosuvastatin vs. placebo	1.23 (1.01–1.50)	0.04

The model was adjusted for log-transformed final CK measures.

CK, creatine phosphokinase; CI, confidence interval.

### Meta-analysis of results

A meta-analysis of the observed effects across GoDARTS, CPRD-STAGE, PREDICTION-ADR and JUPITER revealed that overall, Asp247 homozygotes have 1.34 (95% CI: 1.16–1.54) times the odds of having outcomes associated with statin intolerance compared with carriers of the 247Gly variant (*P-*value 7 × 10^−5^) (*Figure 1*).

## Discussion

We report that the *LILRB5* Asp247 homozygous genotype is associated with increased risk of outcomes associated with statin intolerance across observational, clinically adjudicated and clinical trial datasets. We observe a consistent relationship of increased risk of statin intolerance and the *LILRB5* Asp247 genotype using two definitions of intolerance in the GoDARTS population. There was congruent association with adjudicated cases and controls of SIM in the PREDICTION-ADR study and with myalgia in the JUPITER trial, but no significant association in the CPRD-STAGE study.

A possible mechanism for the role of *LILRB5* in muscle pathology is suggested by a recent study by Kuswanto *et al.*^27^ who highlight a role for the immune system in the repair and regeneration of skeletal muscles. They report that the presence and rapid accumulation of T regulatory (Treg) cells is crucial in the repair of damaged skeletal muscles. It is also reported that statins increase both the number and suppressive function of CD4+ Foxp3+ Treg cells^28^; Foxp3 is a transcription factor that is the master regulator of Treg immune-suppressive activity.^29^ The same study shows sustained increased expression of Foxp3 with statin use. Therefore, mechanisms that induce Foxp3 expression and sustain Treg function are of great interest in understanding muscle homeostasis. We examined mRNA expression data from the GTEx portal and found that the *LILRB5* Asp247 variant is associated with *FOXP3* mRNA expression in the spleen (see Supplementary material online, *Figure S6*).^30^^,^^31^*FOXP3* lies on the X chromosome, indicating a *trans-*eQTL effect, with the *LILRB5* variant having an indirect immunomodulatory effect on *FOXP3* expression. These findings underscore a role for immunogenetics in understanding muscle damage and repair, with *LILRB5* Asp247Gly being the first candidate to be found for common statin intolerance. An illustration of the potential mechanism is provided in Supplementary material online, *Figure S7*.

Previously proposed mechanisms for SIM include an association between statins and reduced mitochondrial function, attenuated energy production, and altered muscle protein degradation contributing to muscle symptoms.^32^^,^^33^ Studies have shown evidence of structural perturbations in skeletal muscle cells associated with statin use.^34^^,^^35^ However, other studies failed to observe such abnormalities.^36^ There are other known immune-mediated forms of statin-related myopathies such as idiopathic inflammatory myositis or immune-mediated necrotizing myopathy, however these are very rarely observed.^37^^,^^38^ Overall, however, there remains a lack of clarity about the underlying pathophysiology of statin intolerance.^18^

While the current variant results in an amino acid substitution, it is not clear that the phenotype results from this protein change. This is especially true as this variant is also associated with *LILRB5* expression in certain tissues in the GTEx portal database (see Supplementary material online, *Figure S8*).^30^^,^^31^ We used GTEx to identify the strongest *cis*-eQTLs for *LILRB5* expression, and found SNPs rs1408812 and rs3852892 showed much stronger effects in skeletal muscles and whole blood, respectively (see Supplementary material online, *Figures S9* and *S10*). However, these polymorphisms have no known associations with CK, LDH or statin intolerance and were not in linkage with the Asp247Gly variant. This demonstrates that the observed phenotypes are not associated with genetically driven variation in *LILRB5* expression and therefore the association with the Asp247Gly variant would appear to be mediated by a functional change in the protein due to the amino acid substitution. However, this has to be confirmed by direct experimentation.

Previous studies have shown that CK levels were associated with *LILRB5* variant^12^^,^^13^ and with all definitions of intolerance, including diagnoses of myalgia, making it a potential confounder. We have ruled out any artefactual associations by including CK measures as a covariate in analyses, where appropriate. Since the GSI phenotype in GoDARTS and diagnoses of myopathy in CPRD-STAGE and PREDICTION-ADR were entirely dependent on elevated CK, this adjustment was not possible. Additionally, the findings of this study could be impacted by confounders that were unmeasured in the cohorts used. Indeed, the non-significant finding in CPRD-STAGE might be due to the use of population controls with no available clinical or demographic information, limiting inclusion of important covariates in the analysis.

Another source of bias in non-randomized studies could be drug interactions that are known to increase risk of SIM.^39^ Inhibition of cytochrome P450 (CYP) 3A4 can increase exposure to simvastatin and atorvastatin several fold. On the other hand, inhibition of hepatic influx transporter OATP1B1 by drugs such as gemfibrozil can increase plasma concentrations of all statins.^40^ Due to the increased risk of muscle toxicity with combination therapy,^41^ fibrates are not generally recommended for concurrent use with statins for primary lipid control,^42^ but are co-prescribed to patients with T2D to control triglyceride levels. Since GoDARTS is primarily a population of T2 Diabetics, fibrates are widely prescribed in the study population. We observe comedications, especially fibrates consistently increased the risk of statin intolerance. However, in adjusted models, the association of the *LILRB5* Asp247Gly variant was independent of the effect of these co-medications. Additionally, the effect is observed in the RCT setting, which confirms that the results from our observational data are not biased.

This finding, in addition to other previously reported genetic associations can facilitate the development of gene risk scores for the prevention of adverse outcomes to statin therapy. Preliminary analyses in GoDARTS reveal a significant interaction between the known *SLCO1B1* polymorphisms and *LILRB5* Asp247Gly (see Supplementary material online, *Results S4*). This would suggest that we are making progress in dissecting 3the complex genetic architecture of statin intolerance, but future studies would be required to examine the corresponding pharmacokinetics of specific statins such as rosuvastatin that are not affected by *SLCO1B1*.

The JUPITER trial allowed us to examine the association of the *LILRB5* Asp247Gly variant in the presence and absence of statins, revealing that homozygosity of Asp247 is associated with increased odds of myalgia regardless of statin allocation. This supports the concept that *LILRB5* Asp247 homozygous genotype modulates CK and LDH levels through statin-independent muscle damage. The association of 247Gly carriers with the development of myalgia in JUPITER is suggestive of a more complex biological gene–drug interaction. The observation that 247Gly carriers showed statin-specific myalgia, suggests a subpopulation of individuals who are inherently protected from myalgia, are susceptible to true ‘statin-induced’ myalgia. In observational data such as GoDARTS it is impossible to determine if intolerance to statins is occurring due to statin-specific or non-specific side effects. However, occurrence of intolerance is associated with increased risk of adverse CV outcomes (see Supplementary material online, *Methods S2*). We believe a recruit-by-genotype trial would be the ideal platform to examine the statin-dependency by *LILRB5* Asp247Gly genotypes, and to further explore the underlying immune mechanisms.

Clinical trials have consistently found no evidence of statin-specific myalgia, just as there is no difference in incidence of myalgia between placebo and rosuvastatin arms of the JUPITER trial.^17^^,^^24^ The lack of association of statins with muscle pain in RCTs has led to a debate regarding the existence of statin-related muscle symptoms. Indeed the difficulty of ascribing causality in statin related muscle symptoms is highlighted by the Goal Achievement after Utilizing an anti-PCSK9 Antibody in Statin-Intolerant Subjects -3 (GAUSS-3) trial.^43^ Data from this trial were the first systematic evaluation of statin-specific myalgia with rechallenge and provided an estimate of 43% of individuals having statin-specific myalgia, and also demonstrated that 37% of intolerant individuals have statin-independent or non-specific myalgia. The *LILRB5* Asp247Gly genotype presents a unique opportunity to probe the immune mechanisms behind the phenomena of muscle pain specific to statins compared to ‘constitutive’ muscle pain that appears in *LILRB5* Asp247 homozygotes.

## Supplementary material

Supplementary material is available at *European Heart Journal* online.

## Supplementary Material

Supplementary Methods
