# Supplementary material for: A common missense variant of LILRB5 is associated with statin intolerance and myalgia
Source: Eur Heart J. 2017 Aug 29;38(48):3569–75. doi: 10.1093/eurheartj/ehx467 (PMC5837247; doi:10.1093/eurheartj/ehx467)
Supplement: Supplementary Methods [file supplementary_lilrb5_ehx467.docx]

Supplementary data
Title: A common missense variant of *LILRB5* is associated with statin intolerance and myalgia
Authors: Siddiqui *et al.*

# Supplementary Methods 1:

## Statin prescribing patterns used to define statin intolerance in GoDARTS

#### Average daily dose

In order to compare dosages of statins – we converted each statin to its equivalent dose of simvastatin ^1^. Average daily dose was calculated by dividing the sum of the product of the usage directions (e.g. 1/d, 2/d etc.) and the strength of the individual tablets (e.g. 10 mg, 20 mg etc.) for each prescription by the total number of days of statin prescription coverage. The average daily dose was 32 mg (of simvastatin or equivalency of other statins), therefore 40 mg was used as the threshold for above average daily dose tolerance.

$Average daily dose =\{\frac{\Sigma\left( Number of statin doses prescribed daily \right)*(mg per dose)}{Total number of days of statin coverage}$*}*

#### Statin switching or discontinuation

A pattern of switching between lipophilic statins (atorvastatin and simvastatin) and hydrophilic statins (pravastatin and rosuvastatin) ^2^, is common when patients suffer adverse effects ^3,4^. The “trial and error” method for locating the ideal statin, is recommended by the European Society of Cardiology (ESC) guidelines ^5^. This pattern was observed in GoDARTS. Statin switching was ascertained in our data by first classifying all the brand name drugs into their generic formulations e.g. simvastatin, atorvastatin, and then looking for changes in the statins prescribed over time. A systemic switch was noted from simvastatin to atorvastatin when the latter came off patent in the UK in 2012. Since this was independent of intolerance, users with such a switch were classifiable as controls, if they met other criteria. We observed that less than 10% of statin users in GoDARTS had switched 3 or more times, and this threshold was applied to account for any re-challenges to statin therapy following reports of adverse reactions ^6–8^. Discontinuation was defined as there being no encashed statin prescription in the period of 9 months (or longer) preceding an individual’s date of death, the study censor date (30^th^ June 2014) or the date they left the data catchment area.

#### Percent Daily Coverage

Percent Daily Coverage (PDC) was computed by calculating the time between the first and last documented statin prescription, calculating the number of days of coverage each prescription provided depending on directions (e.g. 1/day or 2/day) and the quantity of tablets dispensed. The PDC was then calculated by comparing the days of coverage provided by cumulative prescriptions and the total time spent on statin treatment. The average PDC in the study was 89%, therefore 90% was used at the threshold for good adherence.

*PDC*$=\{\frac{\Sigma(days of coverage provided by cumulative statin prescriptions)/(prescription directions )}{Total number of days of statin coverage}$*} x 100*

#### Co-medications

ESC guidelines stipulate that those on interacting comedications are at higher risk of having adverse on-statin outcomes^5^. We examined the data for commonly prescribed co-medications. The most frequently orally administered co-medications were amlodipine (38% of statin users), omeprazole (32%) clopidogrel (17%), and fibrates (5%). All drugs that included warnings or were contra-indicated for concomitant use with statins by the British National Formulary were classified as potentially interacting co-medications, and were included as a covariate in the analyses ^9^.

#### Creatine phosphokinase

CK tests from wards such as Accidents & Emergencies (A&E), cardiac care, stroke, surgical wards and high dependency units were excluded. Individuals with a history of thyroid disease, or those who suffered myocardial infarctions, had kidney disorders or hospital admissions associated with accidents in the 6 months preceding the CK test result were also excluded. Usable test results were categorized into normal: 120 IU/L for women and 180 IU/L for men (NHS Tayside Biochemistry Meta-Data) and above the upper limit of normal (ULN). To define intolerance, the first high CK test result while on statins or within 3 months of collection of the last statin prescription was used. For tolerant controls, the on-statin CK test result most proximate to the study censor date was used, since that would be considered the time of their classification as a control.

## Genotype data for GoDARTS and JUPITER

Blood samples taken at the time of recruitment to GoDARTS were used for DNA extraction and genotyping. Genotype information for the *LILRB5* variant (rs12975366) was obtained for 5785 individuals in GoDARTS. We observed 2295 homozygous for the reference allele (Asp247Asp:T/T), 2715 heterozygotes (Asp247Gly:T/C) and 790 homozygous carriers of the minor allele (Gly247Gly:C/C). In GoDARTS, Genotype data for the *LILRB5* variant (rs12975366) was imputed from Illumina HumanOmni Express -12VI platform (Illumina, San Diego) and Affymetrix 6.0 platform (Affymetrix, Santa Clara) with an imputation quality of 86.8% and 81.7% respectively. A quality threshold of 90% was applied. Imputation was performed against 1000G Phase I V3 reference panel using Impute2 ^10^. Additional samples were genotyped using TAQMAN. Genotyping for 8749 JUPITER trial participants of European ancestry was performed using the Omni1-Quad platform (Illumina, San Diego). The R^2^ of the *LILRB5* Asp247Gly variant, rs12975366 was 0.95 and MAF was 0.40.

# Supplementary Methods 2: Validation of GODARTS phenotypes

## Using *SLCO1B1* genotype risk score

Variants in *SLCO1B1*, encoding the efflux transporter OATP1B1 *,* rs4149056 (Val174Ala) and rs2306283 (Asp130Asn) have been shown to be associated with statin induced myopathy and more general forms of statin intolerance ^11,12^. Using the genotype risk score created by Donnelly *et al.* that was shown to be associated with SI^12^, we attempt to validate our phenotypes. The results are presented in Supplementary Table 1 and show that both phenotypes created are strongly associated with the genotype risk score, where those classified as cases appear to carry the deleterious genotypes.

Supplementary Table 1 Phenotype validation using *SLCO1B1* gene risk score

| **Phenotype** | ***SLCO1B1* gene risk score** | | **Number  of individuals** |
| --- | --- | --- | --- |
|  | **Beta (SE)** | ***P*-value** |  |
| B: Raised CK + non-adherence | 0.48 (0.18) | 0.009 | 944 |
| G: Low Dose intolerance | 0.56 (0.17) | 0.0012 | 1034 |

## Validation of phenotypes using outcome of Major Adverse Cardiovascular Events (MACE)

We performed a survival analysis using the outcome as MACE and classifications of statin intolerance based on our phenotypes of statin intolerance. These analyses were stratified by whether statins were used for primary of secondary prevention of cardiovascular disease (CVD). Statin start date was used as the index date, the occurrence of MACE was classed as outcome, and individuals not having a MACE at end of follow up were censored.

Supplementary Table 2. Number of events of statin failure in each phenotype of statin intolerance

| Phenotype groups | Number of events | Percentage of events (%) |
| --- | --- | --- |
| Cases: GSI | 134 | 41 |
| Controls: GSI | 152 | 30 |
| Cases: LDI | 199 | 42 |
| Controls: LDI | 104 | 31 |

GSI: general statin intolerance with raised CK, LDI: *lowest approved daily starting dose statin intolerance*

Supplementary Table 3. Hazards of statin failure for each phenotype of intolerance. All main effects models were adjusted for age at start of therapy, sex and whether the therapy was for the primary or secondary prevention of CVD. Stratified results are presented for those who were on statins for primary or secondary prevention of CVD.

| **Phenotypes** | **Hazards of Statin Failure** | **Standard Error** | ***P*-value** |
| --- | --- | --- | --- |
| **GSI** | 1.9 | 0.14 | <0.0001 |
| Primary | 2.30 | 0.19 | <0.0001 |
| Secondary | 1.50 | 0.22 | 0.07 |
| **LDI** | 1.9 | 0.14 | <0.0001 |
| Primary | 2.1 | 0.2 | 0.0001 |
| Secondary | 1.6 | 0.2 | 0.028 |

GSI: general statin intolerance with raised CK, LDI: *lowest approved daily starting dose statin intolerance*


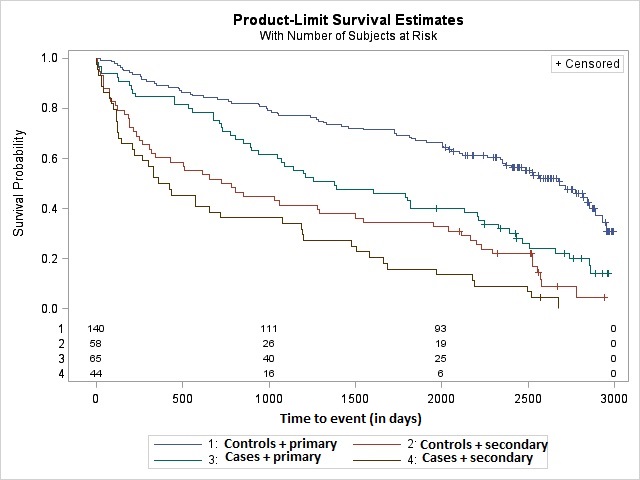


Supplementary Figure 1. Kaplan-Meier representing the hazards of MACE based on classification of general statin intolerance (GSI) stratified by statin use for primary or secondary prevention.


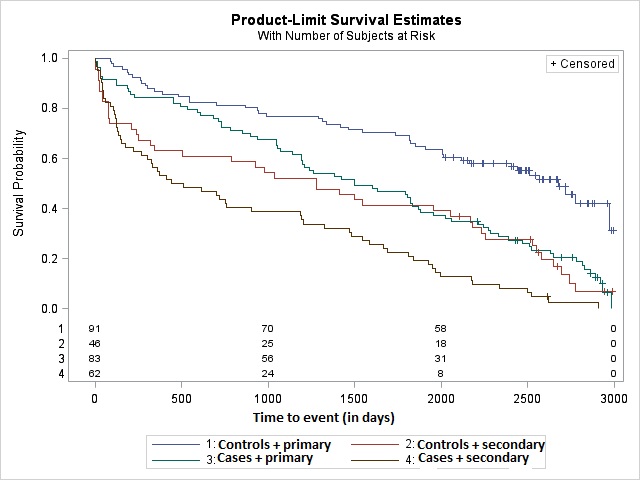


Supplementary Figure 2. Kaplan-Meier representing the hazards of MACE based on classification of lowest approved daily starting dose statin intolerance (LDI) stratified by statin use for primary or secondary prevention.

# Supplementary Methods 3: CPRD-STAGE

## Patient Identification and recruitment

From a cohort of approximately 600,000 patients receiving statins identified in the CPRD (www.cprd.com), a case-control design was used to identify suitable patients for the study as previously described ^13,14^. Participation was restricted to Caucasians ≥18years of age and with the first ever statin prescription at least 1 year after the start of CPRD data collection. Potential cases were selected from the database if they discontinued their implicated statin therapy and demonstrated a rise in CK >4xULN. These were designated "Myopathy". Individuals with a history of rhabdomyolysis or CK >10xULN concurrent to statin exposure were defined as “Severe Myopathy” cases.

All samples were then forwarded onto The University of Liverpool for processing. To preserve anonymity, patient and practice identifier codes were used throughout the recruitment process and all patient contact was via the GP only. A total of 149 myopathy cases were recruited between April 2010 and June 2013 ^13^. Additionally, 5 cases of statin-induced myopathy conforming to our phenotype criteria were identified in the tertiary adult muscle clinic run through Salford Royal NHS Foundation Trust, UK and recruited into the UKMYONET genetic study ^15^. All cases conformed to internationally agreed standards for statin induced myopathy and rhabdomyolysis ^16^.

## Population Control Cohort

Publically accessible genotyping data generated by the Welcome Trust Case Control Consortium (WTCCC) for 2,501 individuals from the UK Blood Service were utilized as a population control in the initial discovery analysis

## Genotyping

At the time of analysis, DNA samples from a total of 135/154 myopathy cases from the discovery cohort were available. At least 1.5 µg DNA from myopathy cases was genotyped for a total of 982,958 SNPs by ARK-Genomics, University of Edinburgh (Edinburgh, UK) using the Illumina OmniExpress Exome v1.0 BeadChip array according to the manufacturers protocol (Illumina Inc, San Diego, CA). The discovery case genotype dataset was merged with the WTCCC dataset prior to SNP phasing using SHAPEIT ^17^ and imputation using IMPUTE2 ^10,18^ was undertaken using 1000 genome phase 3 reference panel. After QC checks 129 cases were available for analysis.

# Supplementary Methods 4: Whole exome sequencing for PREDICTION-ADR cohort

## Library preparation and sequencing

Sequencing of samples was performed using exome-enriched sequence data. SureSelect QXT, XT and XT2 reagents (Agilent Technologies, Wokingham, UK) were used to perform fragmentation, end-repair, A-addition and adaptor ligation reactions to generate Illumina-compatible sequencing libraries respectively in Dundee, Liverpool and Uppsala. Hybridization capture enrichment of whole genome libraries was performed using the SureSelect v5 all-exon probe set, following manufacturer’s recommendations throughout (<http://www.agilent.com/cs/library/usermanuals/Public/G9681-90000.pdf>). Equimolar aliquots of 12 or 10 post-enrichment libraries (6 cases and their 6 matching controls) were pooled before sequencing using version 2 TruSeq chemistry on a Nextseq500 or Hiseq2500 (Illumina Inc., San Diego, CA, USA).

## Read mapping and variant calling

Paired-end 150-bp sequence reads were then analysed through Basespace app BWA enrichment v2.1.0 (Illumina) and Liverpool centre in-house pipeline. The first one gathers demultiplexing, alignment, duplicate removal, variant discovery and annotation. The core algorithm in this workflow is the alignment to indexed reference genome (hg19) using BWA-MEM and indel realignment, base quality score recalibration as well as variant discovery were performed using Genome Analysis Toolkit (GATK) v.1.6 UnifiedGenotyper. The second starts after demultiplexing (from fastq files) and gathers trimming (adaptors, low quality reads) using Cutadapt, alignment using BWA-MEM, duplicate removal using Picard v1.119, variant discovery using Genome Analysis Toolkit (GATK) v.1.6 HaplotypeCaller and annotation using Annovar.

## Data analysis and quality control

Sequencing harmonization was performed across the study centres to ensure comparability of results. Processed sequence data were required to achieve a sequence coverage of at least 20x for more than 70% on targeted bases (Supplementary Table 4). Samples with more than 600 singletons were excluded from analysis. Variant calls were evaluated based on per-sample properties: novel and known variant counts, Ts/Tv ratio, Het/Hom ratio, and deletion/insertion ratio. Sample metrics were compared between the 3 different centres and no significant deviation of calls or samples were noted with 99.8% genotype concordance.

Supplementary Table 4: Sequencing alignment and variant calling characteristics

| **Center** | **Library protocol** | **Sequencer** | **Multiplex per lane** | **Total number of reads** | **% mapped (with duplicates)** | **% at 20x coverage** | **% on target reads** | **% duplicates** | **Number of variants (SNP+INDELs)** |
| --- | --- | --- | --- | --- | --- | --- | --- | --- | --- |
| **Dundee** | SureSelect QXT | NextSeq 500 | 12 | 62,501,759 | 99.62 | 90.37 | 86.27 | 13.12 | 42,606 |
| **Liverpool** | SureSelect XT | HiSeq 2500 | 10 | 71,055,274 | 99.76 | 91.05 | 86.1 | 18.6 | 41,929 |
| **Uppsala** | SureSelect XT2 | HiSeq 2500 | 10 | 62,396,836 | 99.44 | 95.08 | 75.44 | 14.61 | 44,660 |

# Supplementary Results 1: Use of *LILRB5* Asp247Gly variant as dominant

We tested the association between the genotype and mean CK levels an additive and dominant model. The beta estimates in the additive model was lower compared to dominant model (β = -0.026 and -0.027 respectively). Further, when comparing the two homozygous groups Asp247Asp and Gly247Gly, the β = -0.029, not -0.052 as would be predicted by the additive model. This sub-additivity of the effect is observed in both of the other GWAS of CK levels. ^19,20^ The use of a dominant model is slightly underweighting the effect of individuals homozygous for the minor allele; however, in order to perform a case control analyses on an outcome as infrequent as statin intolerance and myalgia, and even more infrequent statin-induced myopathy, we were under-powered to detect meaningful effects in the rare homozygotes. The collapsing of the rare allele into a “carriers v. non-carrier” group is common practice in order to reduce the volatility of small cell sizes in case control analyses.


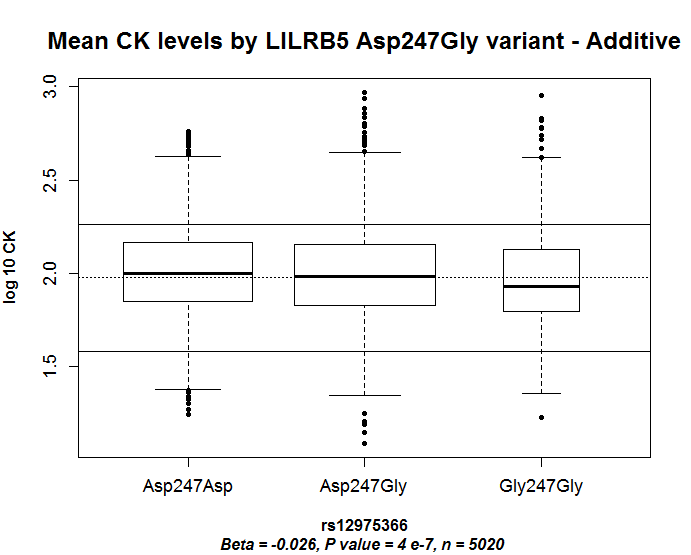


Supplementary Figure 3. Boxplot of mean creatine phosphokinase levels by *LILRB5* Asp247Gly genotypes represented as an additive model. Asp247Asp n = 1972, Asp247Gly n = 2361, Gly247Gly = 688. The upper and lower limits of normal CK measures are represented by the solid lines, while population median CK is represented by the dotted line.


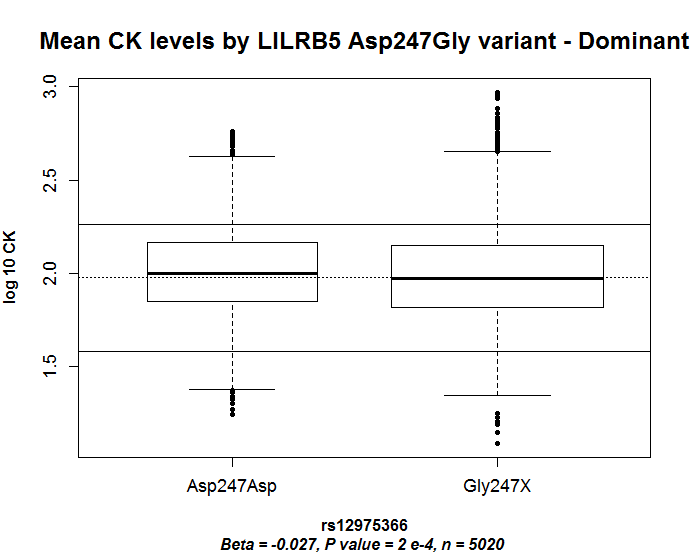


Supplementary Figure 4. Boxplot of mean creatine phosphokinase levels by *LILRB5* Asp247Gly genotypes presented as a dominant model. Asp247Asp n = 1972, Gly247X n = 3057. The upper and lower limits of normal CK measures are represented by the solid lines, while median CK is represented by the dotted line.

# Supplementary Results 2: JUPITER trial data

Supplementary Table 5. Incidence of myalgia by *LILRB5* Asp247Gly genotype

| **Myalgia** | **Asp247 (T/T)** | **247Gly (T/C or C/C)** | **Total** |
| --- | --- | --- | --- |
| No | 2674 | 5238 | 7912 |
| Yes | 301 | 536 | 837 |
| Total | 2975 | 5774 | 8749 |

## Survival analysis in the JUPITER trial, of myalgia and treatment-limiting myalgia by *LILRB5* Asp247Gly genotype

Myalgia was diagnosed as described in the main text. Treatment-limiting myalgia is defined as a combination of myalgia and non-compliance. Non-compliance was defined using a combination of pill counts and non-trial statin use ^21^. Results are presented in Supplementary Table 5 and Supplementary Figure 5.

Supplementary Table 6. Results of cox regressions for myalgia and treatment-limiting myalgia in the JUPITER trial

| **Variables** | **Hazard Ratio (95% CI) of myalgia** | ***P value*** | **Hazard Ratio (95% CI) of treatment-limiting myalgia** | ***P value*** |
| --- | --- | --- | --- | --- |
| **Asp247Asp v. Gly247X** | 1.24 (1.02, 1.52) | 0.039 | 1.32 (0.89, 1.96) | 0.17 |
| **Rosuvastatin** | 0.95 (0.725, 3.24) | 0.63 | 1.05 (0.68, 1.60) | 0.84 |
| **Interaction** | 0.79 (0.60, 1.04) | 0.10 | 1.14 (0.66, 1.97) | 0.64 |


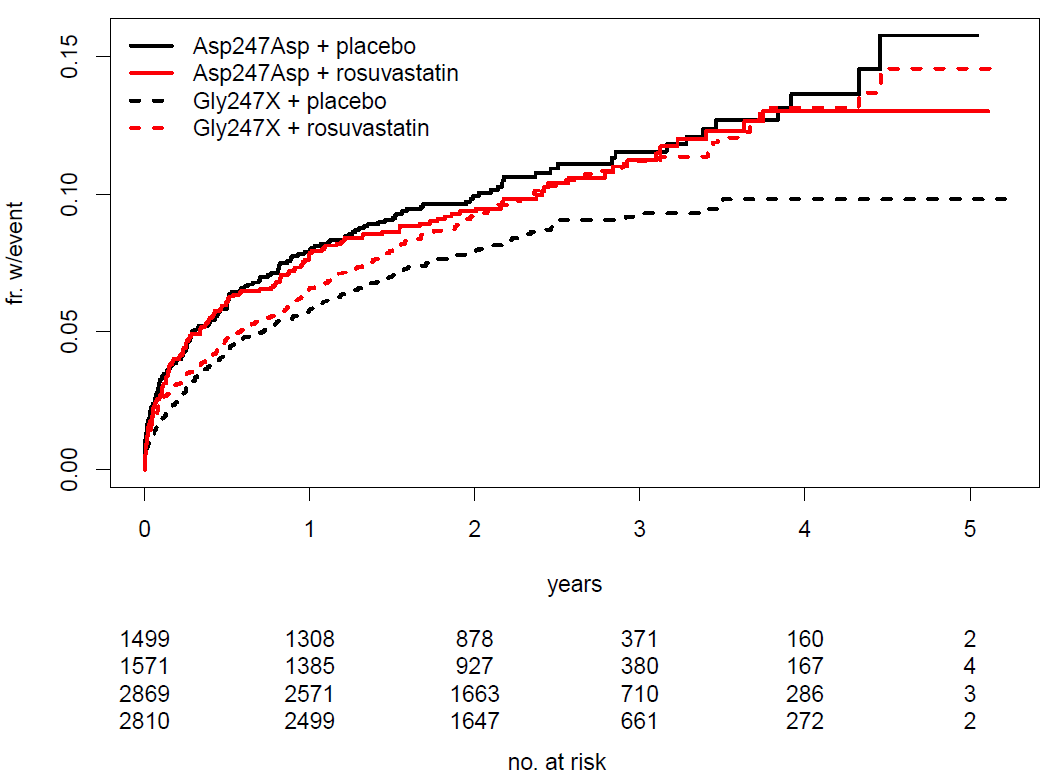


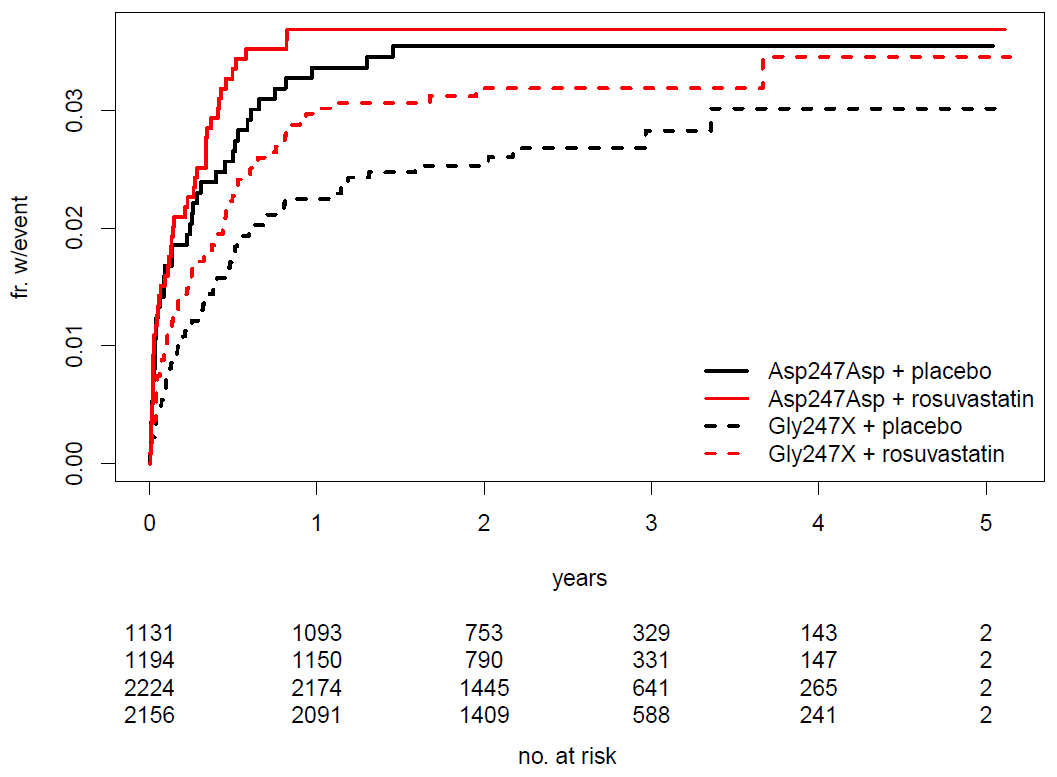


Supplementary Figure 5. Kaplan-Meier of the association of myalgia (upper panel) and treatment-limiting myalgia (lower panel) with *LILRB5* Asp247Gly in JUPITER. The plot represented is not adjusted for final CK levels. While the results for treatment-limiting myalgia are not significant, they do show the stratification of risk that would be expected in the first 2 years of the trial (median follow-up of trial 1.9 years). Risk in descending order: Asp247 homozygotes randomized to receive rosuvastatin > Asp247 homozygotes randomized to receive placebo > Gly247 carriers randomized to receive rosuvastatin > Gly247 carriers randomized to receive placebo.

# Supplementary Results 3: *­*expression Quantitative Trait Loci effects

## eQTL effect of *LILRB5* Asp247Gly on Foxp3 expression in the spleen ^22,23^


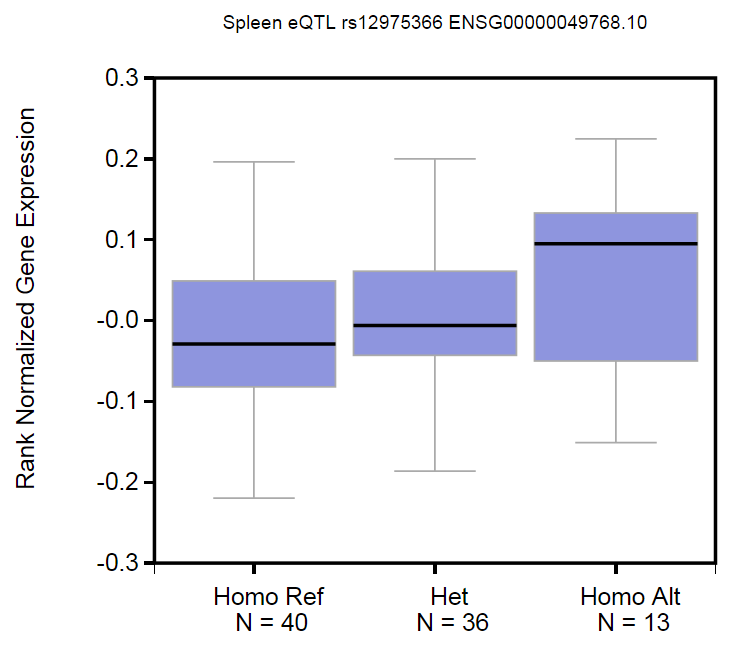


Supplementary Figure 6. Box plot showing the *trans ­*eQTL effect of *LILRB5* Asp247Gly on Foxp3 expression in the spleen (β = 0.31, SE = 0.13, *P-*value = 0.02)

## Illustrative figure of the potential mechanism of *LILRB5* in the repair and regeneration of skeletal muscle cells


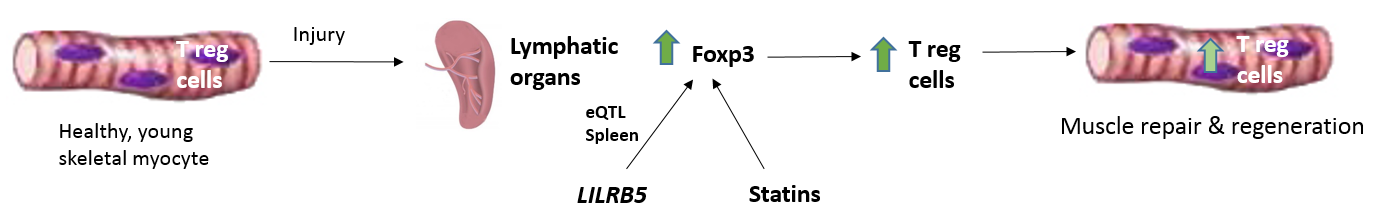


Supplementary Figure 7. Illustration outlining the potential mechanism for *LILRB5* and statin involvement in muscle repair and regeneration. Treg cells are crucial in the repair and regeneration of muscle cells. Foxp3 is a transcription factor that regulates Treg cell immune-suppressive activity. Those with the *LILRB5* Asp247 (risky) genotype have reduced *FOXP3* expression in the spleen compared to those with Gly247X genotype. Statins also have been reported to have an effect on the number and suppressive function of CD4+Foxp3+Treg cells. This hypothesis will have to be investigated further in detailed recruit by genotype studies.

Foxp3: Forkhead box P3, Treg: T regulator, LILRB5: Leukocyte Immunoglobulin-like Receptor subfamily B

## *Cis*-eQTL effect of Asp247Gly variant on *LILRB5* expression in the spleen


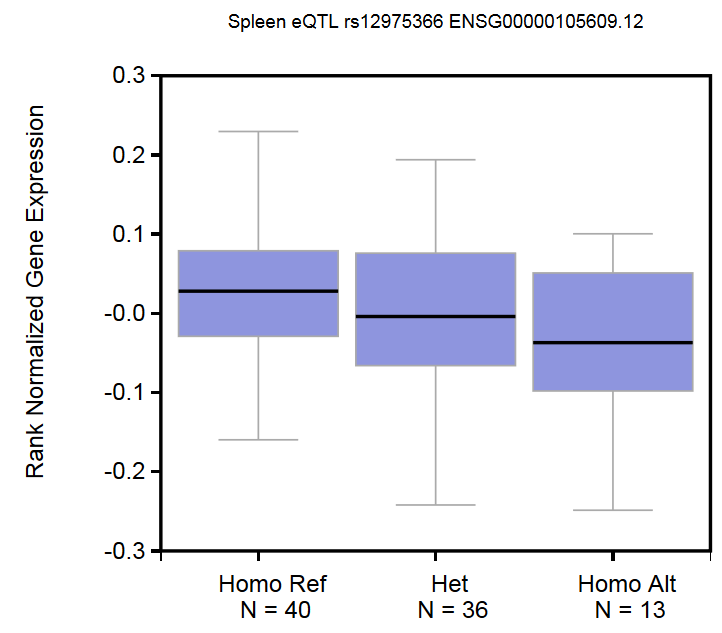


Supplementary Figure 8. Box plot showing the eQTL effect of rs12975366 on *LILRB5* expression in the spleen (β = -0.28, SE = 0.13, *P-*value = 0.04).

## Strongest eQTL for *LILRB5* in whole blood: rs3852892 ^22,23^


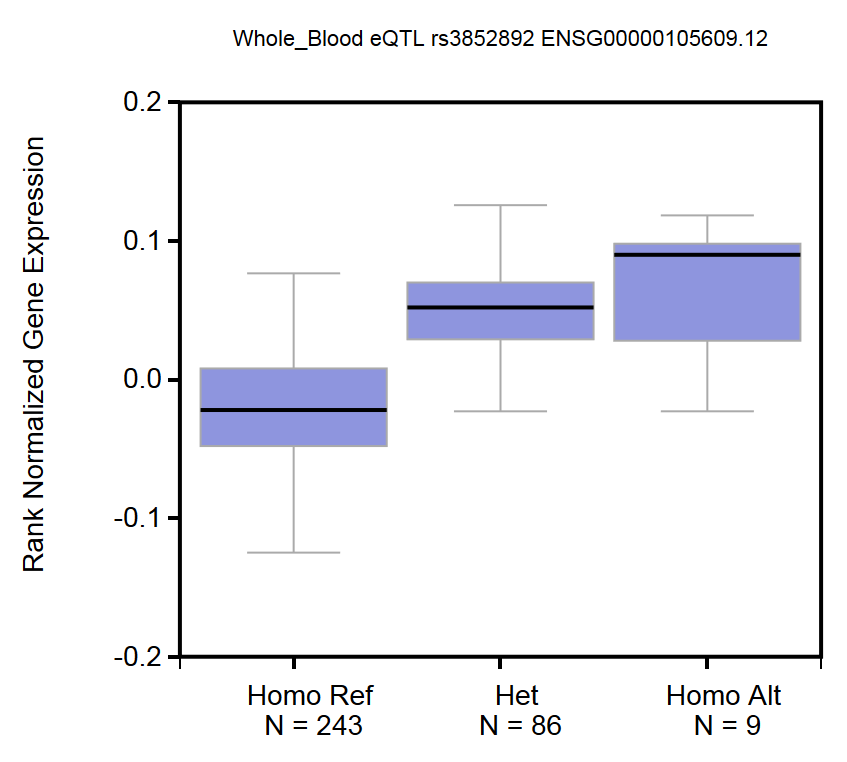


Supplementary Figure 9. Box plot showing the eQTL effect of rs3852892 on *LILRB5* expression in whole blood (β = 0.98, SE = 0.07, *P-*value = 8x10^-36^).

## Strongest eQTL for *LILRB5* in the spleen: rs1408812 ^22,23^


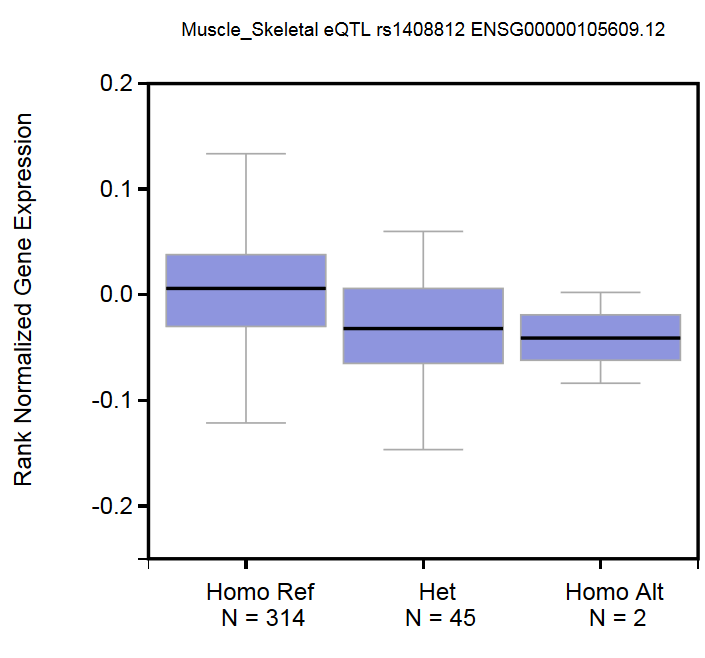


Supplementary Figure 10. Box plot showing the eQTL effect of rs1408812 on *LILRB5* expression in skeletal muscle (β = -0.39, SE = 0.08, *P-*value = 7x10^-6^).

# Supplementary Results 4: Interaction between *SLCO1B1 genotypes and LILRB5 Asp247Gly*

Both the *SLCO1B1* diplotype and the *LILRB5* Asp247Gly genotype were placed in a model together. Both were significantly associated with General Statin Intolerance (GSI) and Low Dose Intolerance (LDI). Interestingly, the beta estimate for the *LILRB5* genotype was higher after adjusting for the *SLCO1B1* diplotype. In addition, a significant interaction term was observed for these genotypes for both intolerance groups. The interaction term can be interpreted as the increased risk being specifically seen in those individuals with the risk alleles at both *SLCO1B1* and *LILRB5.* However this needs to be confirmed in larger populations

Supplementary Table 7: Results of models adjusted for *SLCO1B1* genotypes and *LILRB5* Asp247Gly

| Phenotype\Genotype | SLCO1B1 diplotype | LILRB5 genotype | Interaction term | |
| --- | --- | --- | --- | --- |
|  | OR (95% Confidence Interval) | | β estimate (Standard Error) | OR (95% Confidence Interval)* |
| GSI: GoDARTS | 1.59 (1.10, 2.28) *P = 0.013* | 2.08 (1.09, 3.98) *P = 0.027* | 0.61 (0.24)  *P = 0.012* | 1.84 (1.15, 2.95) |
| LDI: GoDARTS | 1.73 (1.23, 2.43) *P = 0.0016* | 2.09 (1.12, 3.89) *P = 0.02* | 0.47 (0.21) *P = 0.02* | 1.60 (1.06, 2.41) |

*GSI: general statin intolerance with raised CK, LDI: lowest approved daily starting dose statin intolerance
*Calculated from linear regression output*

# Supplementary references

1. Maron DJ, Fazio S, Linton MF. Current perspectives on statins. *Circulation* 2000;**101**:207–213.

2. Schachter M. Chemical, pharmacokinetic and pharmacodynamic properties of statins: an update. *Fundam Clin Pharmacol* England; 2005;**19**:117–125.

3. Stroes ES, Thompson PD, Corsini A, Vladutiu GD, Raal FJ, Ray KK, Roden M, Stein E, Tokgozoglu L, Nordestgaard BG, Bruckert E, Backer G De, Krauss RM, Laufs U, Santos RD, Hegele RA, Hovingh GK, Leiter LA, Mach F, Marz W, Newman CB, Wiklund O, Jacobson TA, Catapano AL, Chapman MJ, Ginsberg HN. Statin-associated muscle symptoms: impact on statin therapy-European Atherosclerosis Society Consensus Panel Statement on Assessment, Aetiology and Management. *Eur Heart J* England; 2015;**36**:1012–1022.

4. European Association for Cardiovascular Prevention & Rehabilitation, Reiner Z, Catapano AL, Backer G De, Graham I, Taskinen M-R, Wiklund O, Agewall S, Alegria E, Chapman MJ, Durrington P, Erdine S, Halcox J, Hobbs R, Kjekshus J, Filardi PP, Riccardi G, Storey RF, Wood D, ESC Committee for Practice Guidelines (CPG) 2008-2010 and 2010-2012 Committees. ESC/EAS Guidelines for the management of dyslipidaemias: the Task Force for the management of dyslipidaemias of the European Society of Cardiology (ESC) and the European Atherosclerosis Society (EAS). *Eur Heart J* 2011;**32**:1769–1818.

5. Piepoli MF, Hoes AW, Agewall S, Albus C, Brotons C, Catapano AL, Cooney MT, Corr?? U, Cosyns B, Deaton C, Graham I, Hall MS, Hobbs FDR, L??chen ML, L??llgen H, Marques-Vidal P, Perk J, Prescott E, Redon J, Richter DJ, Sattar N, Smulders Y, Tiberi M, Worp HB Van Der, Dis I Van, Verschuren WMM, Binno S, Backer G De, Roffi M, Aboyans V, et al. 2016 European Guidelines on cardiovascular disease prevention in clinical practice. *Eur Heart J* 2016;**37**:2315–2381.

6. Joy TR, Hegele RA. Narrative review: statin-related myopathy. *Ann Intern Med* 2009;**150**:858–868.

7. Grundy SM. Can statins cause chronic low-grade myopathy? Ann. Intern. Med. United States; 2002. p. 617–618.

8. Banach M, Rizzo M, Toth PP, Farnier M, Davidson MH, Al-Rasadi K, Aronow WS, Athyros V, Djuric DM, Ezhov M V, Greenfield RS, Hovingh GK, Kostner K, Serban C, Lighezan D, Fras Z, Moriarty PM, Muntner P, Goudev A, Ceska R, Nicholls SJ, Broncel M, Nikolic D, Pella D, Puri R, Rysz J, Wong ND, Bajnok L, Jones SR, Ray KK, et al. Statin intolerance - an attempt at a unified definition. Position paper from an International Lipid Expert Panel. *Arch Med Sci* Poland; 2015;**11**:1–23.

9. Joint Formulary Committee. British National Formulary. London BMJ Gr. Pharm. Press. 2014. http://www.medicinescomplete.com (25 November 2015)

10. Howie BN, Donnelly P, Marchini J. A flexible and accurate genotype imputation method for the next generation of genome-wide association studies. *PLoS Genet* United States; 2009;**5**:e1000529.

11. Link E, Parish S, Armitage J, Bowman L, Heath S, Matsuda F, Gut I, Lathrop M, Collins R. SLCO1B1 variants and statin-induced myopathy--a genomewide study. *N Engl J Med* 2008;**359**:789–799.

12. Donnelly LA, Doney AS, Tavendale R, Lang CC, Pearson ER, Colhoun HM, McCarthy MI, Hattersley AT, Morris AD, Palmer CN. Common nonsynonymous substitutions in SLCO1B1 predispose to statin intolerance in routinely treated individuals with type 2 diabetes: a go-DARTS study. *Clin Pharmacol Ther* 2011;**89**:210–216.

13. O’Meara H, Carr DF, Evely J, Hobbs M, McCann G, Staa T van, Pirmohamed M. Electronic Health Records For Biological Sample Collection: Feasibility Study Of Statin-Induced Myopathy Using The Clinical Practice Research Datalink. *Br J Clin Pharmacol* 2013;

14. Staa TP van, Carr DF, O’Meara H, McCann G, Pirmohamed M. Predictors and outcomes of increases in creatine phosphokinase concentrations or rhabdomyolysis risk during statin treatment. *Br J Clin Pharmacol* England; 2014;**78**:649–659.

15. Jani M, Massey J, Wedderburn LR, Vencovsky J, Danko K, Lundberg IE, Padyukov L, Selva-O’Callaghan A, Radstake T, Platt H, Warren RB, Griffiths CE, Lee A, Gregersen PK, Miller FW, Ollier WE, Cooper RG, Chinoy H, Lamb JA. Genotyping of immune-related genetic variants identifies TYK2 as a novel associated locus for idiopathic inflammatory myopathies. Ann. Rheum. Dis. England; 2014. p. 1750–1752.

16. Alfirevic A, Neely D, Armitage J, Chinoy H, Cooper RG, Laaksonen R, Carr DF, Bloch KM, Fahy J, Hanson A, Yue QY, Wadelius M, Maitland-van Der Zee AH, Voora D, Psaty BM, Palmer CN, Pirmohamed M. Phenotype Standardization for Statin-Induced Myotoxicity. *Clin Pharmacol Ther* 2014;

17. Delaneau O, Coulonges C, Zagury J-F. Shape-IT: new rapid and accurate algorithm for haplotype inference. *BMC Bioinformatics* England; 2008;**9**:540.

18. Howie B, Marchini J, Stephens M. Genotype imputation with thousands of genomes. *G3 (Bethesda)* United States; 2011;**1**:457–470.

19. Dube MP, Zetler R, Barhdadi A, Brown AM, Mongrain I, Normand V, Laplante N, Asselin G, Zada YF, Provost S, Bergeron J, Kouz S, Dufour R, Diaz A, Denus S de, Turgeon J, Rheaume E, Phillips MS, Tardif JC. CKM and LILRB5 are associated with serum levels of creatine kinase. *Circ Cardiovasc Genet* 2014;**7**:880–886.

20. Kristjansson RP, Oddsson A, Helgason H, Sveinbjornsson G, Arnadottir GA, Jensson BO, Jonasdottir A, Jonasdottir A, Bragi Walters G, Sulem G, Oskarsdottir A, Benonisdottir S, Davidsson OB, Masson G, Th Magnusson O, Holm H, Sigurdardottir O, Jonsdottir I, Eyjolfsson GI, Olafsson I, Gudbjartsson DF, Thorsteinsdottir U, Sulem P, Stefansson K. Common and rare variants associating with serum levels of creatine kinase and lactate dehydrogenase. *Nat Commun* England; 2016;**7**:10572.

21. Chasman DI, Giulianini F, MacFadyen J, Barratt BJ, Nyberg F, Ridker PM. Genetic determinants of statin-induced low-density lipoprotein cholesterol reduction: the Justification for the Use of Statins in Prevention: an Intervention Trial Evaluating Rosuvastatin (JUPITER) trial. *Circ Cardiovasc Genet* United States; 2012;**5**:257–264.

22. Human genomics. The Genotype-Tissue Expression (GTEx) pilot analysis: multitissue gene regulation in humans. *Science* United States; 2015;**348**:648–660.

23. Carithers LJ, Moore HM. The Genotype-Tissue Expression (GTEx) Project. Biopreserv. Biobank. United States; 2015. p. 307–308.
